# Efficacy and safety of quadriceps tendon autograft versus bone–patellar tendon–bone and hamstring tendon autografts for anterior cruciate ligament reconstruction: a systematic review and meta-analysis

**DOI:** 10.1186/s10195-024-00801-2

**Published:** 2024-12-18

**Authors:** Xiao-Feng Zhang, Pan Liu, Jun-Wu Huang, Yao-Hua He

**Affiliations:** 1 Department of Orthopedics, Shanghai Sixth People’s Hospital, Jinshan Branch, Shanghai, 201599 China; 2 Department of Orthopedics, Sichuan Academy of Medical Sciences and Sichuan Provincial People’s Hospital, University of Electronic Science and Technology of China, Chengdu, China

**Keywords:** Quadriceps tendon autograft, Bone–patellar tendon–bone, Hamstring tendon autografts, Anterior cruciate ligament reconstruction, Meta-analysis

## Abstract

**Background:**

Quadriceps tendon (QT), bone–patellar tendon–bone (BPTB), and hamstring tendon (HT) autografts are widely used for anterior cruciate ligament reconstruction (ACLR), but the optimal autograft choice remains controversial. This study assessed the treatment effects of QT versus BPTB and HT autografts for ACLR.

**Methods:**

The PubMed, Embase, and Cochrane Library databases were systematically searched for eligible studies published from inception until July 2022. Effect estimates were presented as odds ratios (OR) and weighted mean differences (WMD) with 95% confidence intervals (CI) for categorical and continuous variables, respectively. All pooled analyses were performed using a random-effects model.

**Results:**

Twenty-one studies (3 randomized controlled trials [RCTs], 3 prospective studies, and 15 retrospective studies) involving 2964 patients with ACLR were selected for meta-analysis. Compared with the HT autograft, the QT autograft was associated with a reduced risk of graft failure (OR: 0.46; 95% CI: 0.23–0.93; *P* = 0.031). Compared with the BPTB autograft, the QT autograft was associated with a reduced risk of donor site pain (OR: 0.16; 95% CI: 0.10–0.24; *P* < 0.001). Moreover, the QT autograft was associated with a lower side-to-side difference than that observed with the HT autograft (WMD: − 0.74; 95% CI: − 1.47 to − 0.01; *P* = 0.048). Finally, compared with the BPTB autograft, the QT autograft was associated with a reduced risk of moderate-to-severe kneecap symptoms during sports and work activities (OR: 0.14; 95% CI: 0.05–0.37; *P* < 0.001).

**Conclusions:**

The findings of this study suggest that the QT autograft can be defined as a safe and effective alternative choice for ACLR, but its superiority is yet to be proven by RCTs and prospective studies.

*Level of evidence*

Level III.

**Supplementary Information:**

The online version contains supplementary material available at 10.1186/s10195-024-00801-2.

## Introduction

Anterior cruciate ligament (ACL) injury of the knee joint is the most common injury in young active individuals and is caused by sports activities such as basketball, football, skiing, and volleyball [1, 2]. The ACL and other tissues of the knee joint work synergistically to prevent excessive forward movement of the tibia and maintain normal function of the knee joint [3]. Therefore, joint stability and biomechanics are impacted by a knee with an ACL deficiency [4, 5]. Furthermore, knees lacking a healthy ACL are characterized by altered joint movements, which in turn influence common daily activities such as walking, climbing stairs, descending stairs, and jumping [6, 7]. Moreover, this abnormality can disturb the contact area and increase the magnitude of shear forces at the knee joint, potentially exacerbating the development of osteoarthritis [8, 9].

ACL reconstruction (ACLR) could lessen changes in knee biomechanics, which, in turn, could improve knee stability and kinematics [10, 11]. There is a substantial connection between loading and degeneration of knee joint cartilage and factors such as the peak external knee adduction moment, the peak internal rotation angle, the medial contact force, and the knee flexion angle [12, 13]. Consequently, the choice of graft in ACLR can significantly impact both the pace and degree of knee rehabilitation [14, 15]. A prior study compared the knee muscle strength achieved at 6 and 12 months after ACLR using bone–patellar tendon–bone (BPTB) and hamstring tendon (HT) autografts and found that the HT autograft was associated with significant deficits in flexor muscle strength [16]. Nowadays, the quadriceps tendon (QT) autograft has increasingly gained favor in clinical settings due to its perceived advantages, including maintenance of hamstring integrity and function, fewer occurrences of anterior knee pain and numbness, a diminished risk of patellar fractures, and minimal bleeding at the bone harvest site [17]. However, the question of what constitutes the optimal autograft choice for ACLR remains unresolved. Therefore, we performed a systematic review and meta-analysis to assess the treatment effects of QT versus BPTB and HT autografts for ACLR.

## Methods

### Literature search and selection criteria

The Preferred Reporting Items for Systematic Reviews and Meta-analysis (PRISMA) guidelines were used to conduct and report this study [18]. Studies comparing the efficacy and safety of QT versus BPTB and HT autografts for ACLR were considered eligible for this study, and the publication language and status were not restricted. The PubMed, EmBase, and Cochrane Library databases were systematically searched for eligible studies published until July 2022. The core search terms included “quadriceps tendon autograft” or “quadriceps graft” and “anterior cruciate ligament reconstruction” or “ACL reconstruction.” Reference lists were manually searched for relevant reviews to identify new studies that met the inclusion criteria.

Two reviewers independently performed the literature search and study selection, and conflicts between the reviewers were resolved through mutual discussion by reviewing the original article until a consensus was reached. Studies were included if they met the following criteria (PICOS: patients, intervention, control, outcomes, and study design): (1) patients—all patients should have undergone ACLR; (2) intervention—QT autograft; (3) control—BPTB or HT autografts; (4) outcomes—knee stability, functional outcome, graft site pain, and graft failure rate; and (5) study design—randomized controlled trials (RCTs) and prospective or retrospective observational studies.

### Data extraction and quality assessment

The following information was independently extracted by the two reviewers: first author’s surname, publication year, study design, country, sample size, mean age, male proportion, mean time to reconstruction, intervention, control, rehabilitation procedures, follow-up, and reported outcomes. These two reviewers independently assessed the quality of the included studies using the risk of bias approach according to the methods described by the Cochrane Collaboration and Newcastle–Ottawa Scale (NOS) for RCTs and observational studies, respectively [19, 20]. The Cochrane Collaboration risk of bias included random sequence generation, allocation concealment, blinding of participants and personnel, blinding of the outcome assessment, incomplete outcome data, selective reporting, and other biases (high quality: low risk for 5–7 items; moderate quality: low risk for 3–5 items; and low quality: low risk for 0–3 items). The NOS included selection, comparability, and outcome domains and used a “star system” with scores ranging from 0–9 (high quality: 7–9; moderate quality: 4–6; and low quality: 0–3). Inconsistent results between reviewers regarding data collection and quality assessment were resolved by discussing the original article with an additional reviewer.

### Statistical analysis

Treatment outcomes were categorized as categorical or continuous data, and odds ratios (OR) or weighted mean differences (WMD) with 95% confidence intervals (CI) were calculated before data pooling. Considering the underlying variations across the included studies, a random-effects model was applied to calculate the pooled effect estimates [21, 22]. The heterogeneity for each outcome was assessed using *I*^2^ and Cochran’s* Q* statistic, and significant heterogeneity was defined as *I*^2^ ≥ 50.0% or Cochran’s *P* < 0.10 [23, 24]. The robustness of the pooled conclusion was assessed using sensitivity analysis based on the sequential exclusion of individual studies [25]. Subgroup analyses were performed to investigate outcomes according to the study design, and the differences among subgroups were assessed using the interaction *P* test [26]. Publication bias for the investigated outcomes was assessed using funnel plots, Egger’s test, and Begg’s test [27, 28]. All reported *P* values for the pooled results were two-sided, and the inspection level was 0.05. Statistical analysis was performed using the STATA software (version 14.0; Stata Corporation, College Station, TX, USA).

## Results

### Literature search

**Fig. 1:**
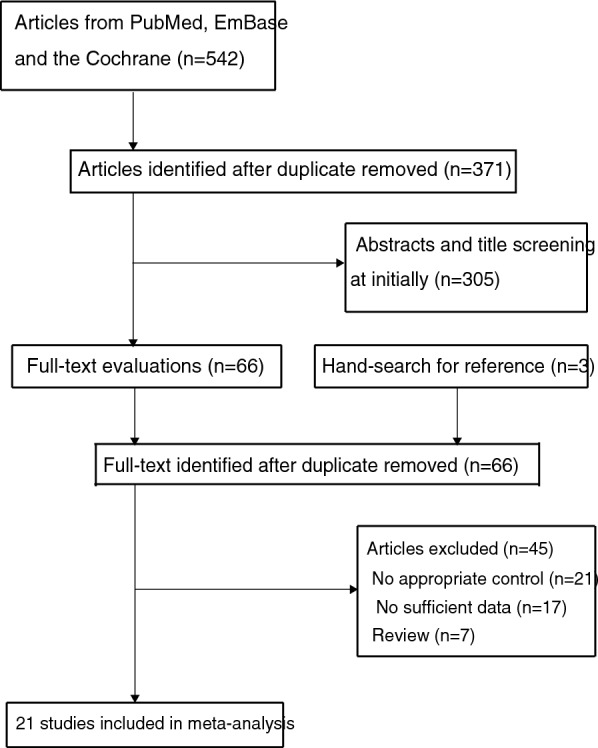
PRISMA flowchart for the literature search and study selection process.* PRISMA* Preferred Reporting Items for Systematic Reviews and Meta-analysis guidelines

A total of 542 articles were identified from electronic searches, and 371 studies were retained after duplicate articles were removed. Subsequently, 305 studies were excluded because their titles and abstracts were irrelevant. The full text of the remaining 66 studies was retrieved for evaluation. Reviewing the reference lists yielded three potential studies for inclusion, which were subsequently included in the electronic searches. Then a total of 45 studies were excluded due to no appropriate control (*n* = 21), no sufficient data (*n* = 17), and the review design (*n* = 7). Finally, 21 studies were selected for the meta-analysis [29–49] (Fig. 1).

### Study characteristics

The baseline characteristics of the included studies and involved patients are shown in Table 1. Of the 21 included studies, 3 were RCTs, 3 were prospective cohort studies, and 15 were retrospective observational studies. Nine studies compared QT with BPTB autografts for ACLR, 11 studies compared QT with HT autografts for ACLR, and one study compared QT with BPTB and HT autografts for ACLR. The included studies involved 2,964 patients who had undergone ACLR, and the sample size for each study ranged from 28 to 875. The methodological quality of the included studies is shown in Table 1. The overall quality of the included studies was moderate to high.

**Table 1:** The baseline characteristics of the included studies and involved patients

Study	Publication year	Study design (evidence level)	Country	Sample size	Age (years)	Male (%)	Mean time to reconstruction	Intervention	Control	Rehabilitation procedures	Follow-up (months)	Quality
Gorschewsky [29]	2007	Retrospective (III)	Switzerland	260 (124/136)	32.0	70.0	1.0 months	QT	BPTB	Patients got a knee brace with the flexion limit at 90° and full extension capacity for 4 weeks postoperatively	24.0	High
Han [30]	2008	Retrospective (III)	Korea	144 (72/72)	27.8	94.4	22.4 months	QT	BPTB	NA	24.0	Moderate
Geib [31]	2009	Retrospective (IV)	USA	221 (191/30)	30.7	51.6	NA	QT	BPTB	Physical-therapist-assisted range-of-motion and closed-chain exercises began on the first postoperative day. Patients used two crutches with weight-bearing as tolerated for the first 3 weeks, followed by a single crutch for the next 3 weeks. Patients began in-line running at 3 months and returned to full activity at 6 months	56.8	Moderate
Kim [32]	2009	Retrospective (IV)	Korea	61 (29/31)	27.2	41.0	6.0 months	QT	BPTB	All patients were permitted immediate partial weight-bearing using crutches. Patients were allowed to bear their full weight approximately 4 weeks after surgery. By the 12th week, jogging, swimming, and cycling were permitted. A return to sports involving jumping, pivoting, or sidestepping was allowed after 6 months	24.0	Moderate
Kim [33]	2009	Retrospective (IV)	Korea	48 (21/27)	28.8	81.3	14.2 months	QT	BPTB	Patients were permitted immediate weight-bearing, as tolerated, and full ROM, especially restoration of full knee extension. Patients wore a knee brace without a flexion limit for 4 weeks. Partial leg extension exercises from 90° to 40°, without resistance, started 2 weeks after surgery. Postoperative rehabilitation goals from weeks 1 to 4 included a return to full ROM, progression to full weight-bearing, and increasing muscle contracture through full ROM. By the fifth week, short-arc quadriceps movements from 0° to 30° were initiated. From 4 to 12 weeks, patients aimed to perform activities of daily living. By the 12th week, jogging, swimming, and cycling were permitted. Sports involving jumping, pivoting, or sidestepping were permitted after 6 months	26.0	Moderate
Sofu [34]	2013	Retrospective (IV)	Turkey	44 (23/21)	27.7	95.5	16.1 months	QT	HT	NA	37.6	Moderate
Kim [35]	2014	Retrospective (III)	Korea	281 (89/158/41); 146 (53/69/24)	30.4/31.3	81.7/86.7	29.3/36.4 months	QT	BPTB, HT	Patients were allowed immediate tolerable weight-bearing and knee motion. After 12 weeks, jogging, cycling, and swimming were permitted. After 6 months, sports activity involving pivoting, jumping, or sidestepping was allowed	24.0	Moderate
Lund [36]	2014	RCT (II)	Denmark	51 (26/25)	30.5	82.4	18.1 months	QT	BPTB	The knee was allowed free range of motion from day 1, followed by isometric quadriceps and passive flexion exercises. Patients were allowed full weight-bearing as tolerated by pain and effusion. Stationary bicycle exercises were used from the fourth postoperative week, and progressive quadriceps strength exercises were allowed from the sixth postoperative week. Running was allowed at 3 months postoperatively, followed by a return to cutting actions and contact sports at 9 months postoperatively or later. Rehabilitation was supervised by a hysiotherapist for 3 months using criteria-based activity progression	24.0	Moderate
Haner [37]	2016	Prospective (III)	Germany	51 (25/26)	35.8	68.6	NA	QT	HT	Partial weight-bearing and a full range of motion were permitted. Crutches were used for 4 weeks. A rehabilitation brace was used for 6 weeks. Closed-chain exercises were started after 2 weeks. If meniscal repair was performed during the same operation, a 0°–60° range of motion of the knee was allowed for the first 6 weeks	24.0	Moderate
Lee [38]	2016	Retrospective (III)	Korea	96 (48/48)	30.5	91.7	NA	QT	HT	Continuous passive motion exercises were started 2 days after the surgery, and they continued for 1 to 2 days while the patient was still hospitalized. Full hyperextension was obtained in 1 week and full flexion in 6 weeks. A motion-controlled brace was worn for 6 weeks. Open kinetic chain exercise was recommended, and kinetic exercises were progressed as tolerated. Only partial weight-bearing was allowed for 6 weeks, and full activity was recommended at 6 months postoperatively	34.9	High
Cavaignac [39]	2017	Retrospective (III)	Switzerland	86 (45/41)	31.5	57.0	11.0 months	QT	HT	Partial weight-bearing (15–20 kg) was allowed for the first 4 weeks, followed by full weight-bearing afterward. The sutures were removed on postoperative day 12. All patients underwent the same rehabilitation regimen. A booklet was given to all patients to ensure that they all followed the standardized rehabilitation regimen	43.2	Moderate
Runer [40]	2018	Prospective (III)	Austria and Germany	80 (40/40)	34.5	57.5	2.6 months	QT	HT	A knee brace limiting flexion up to 90° was used starting on the first postoperative day, and partial weight-bearing was allowed immediately after surgery, with crutches used for at least 2 weeks. Thereafter, full weight-bearing and an unrestricted range of motion was allowed. After a 2-day inpatient stay, patients attended outpatient physical therapy 2–3 times per week for at least 8–12 weeks	24.0	High
Martin-Alguacil [41]	2018	RCT (II)	Spain	51 (26/25)	18.9	76.5	NA	QT	HT	Both groups followed the same pre- and-postrehabilitation protocol based on muscular strength, endurance, and neuromuscular control (24 weeks)	24.0	High
Hunnicutt [42]	2019	Retrospective (III)	USA	30 (15/15)	21.5	63.3	7.5 months	QT	BPTB	NA	8.0	Moderate
Todor [43]	2019	Retrospective (III)	Romania	72 (39/33)	29.7	68.1	NA	QT	HT	Partial weight-bearing with crutches was done for a period of 4 weeks. From 4 weeks on, full weight-bearing was allowed. A stationary bicycle was recommended from 6 weeks on; running on a treadmill as well as swimming were allowed from 3 months on. Sports activities requiring pivoting actions were allowed from 9 months postoperatively	34.0	Moderate
Pennock [44]	2019	Retrospective (IV)	USA	90 (28/62)	14.8	68.9	NA	QT	HT	All patients were allowed immediate motion of the knee and were kept toe-touch weight-bearing for 1 week. Formal therapy was initiated at that time. A running progression was started 3 months after surgery, and patients were allowed to return to sport 6–12 months after surgery if they passed a return-to-sport test	24.0	Moderate
Perez [45]	2019	Retrospective (IV)	USA	50 (28/22)	22.5	72.0	NA	QT	BPTB	All patients were placed in a range-of-motion brace and underwent similar rehabilitation protocols initiated within 1 week of surgery	33.0	Moderate
Vilchez-Cavazos [46]	2020	RCT (II)	Mexico	28 (14/14)	23.0	82.1	NA	QT	HT	The patients were allowed to walk on crutches without weight-bearing during the first 2 weeks, knee flexing was permitted up to 90°, and complete extension was allowed. Weight-bearing was permitted with crutch assistance (weeks 2–4). Knee flexiong was permitted to 120°, and complete extension was allowed (during the first postoperative month). In the second month, closed-chain exercise was initiated, and open-chain exercise was allowed if the patient tolerated it. In the third month, the open- and closed-chain exercises continued, and jogging was permitted. Finally, jogging was completely permitted, and the patients focused on increasing strength and muscle mass (4–6 months); a return to playing sports was permitted (6–8 months)	12.0	High
Runer [47]	2020	Prospective (III)	Austria	875 (217/658)	30.8	60.6	NA	QT	HT	Early improvement of range of motion and pain control, which was originally developed and implemented for patients treated with HT. Patients treated with a QT autograft with bone block were not subject to a more aggressive rehabilitation program. After a 2-day inpatient stay for mobilization training and pain therapy, all patients attended outpatient physical therapy for at least 12 weeks. During the first 2 postoperative weeks, only partial weight-bearing was allowed, and knee flexion was limited to 90° with a knee brace. Thereafter, weight-bearing as tolerated and an unrestricted knee range of motion were allowed	24.0	Moderate
Aslam [48]	2021	Retrospective (III)	India	70 (35/35)	24.8	67.1	6.4 months	QT	HT	NA	12.0	Moderate
Hogan [49]	2021	Retrospective (III)	USA	119 (39/80)	20.2	58.0	NA	QT	BPTB	Patients were fitted with a hinged knee brace, which was to be locked in extension and worn at all times for ambulation and for sleeping. Patients were instructed to be weight-bearing as tolerated with crutches to assist with walking. Home exercises were initiated 24 h after surgery with the goal of complete extension and 90° of flexion at initial follow-up, which was scheduled for 7–10 days postoperatively. Meniscus repair rehabilitation was the same in both groups, with patients either weight-bearing as tolerated or toe-touch weight-bearing based on their tear pattern. Formal physical therapy was initiated after the initial follow-up appointment. The return to greater-level activities was individualized based on standardized, minimum time, and progressive functional rehabilitation criteria	26.5	Moderate

### Graft failure and donor-site pain

**Fig. 2:**
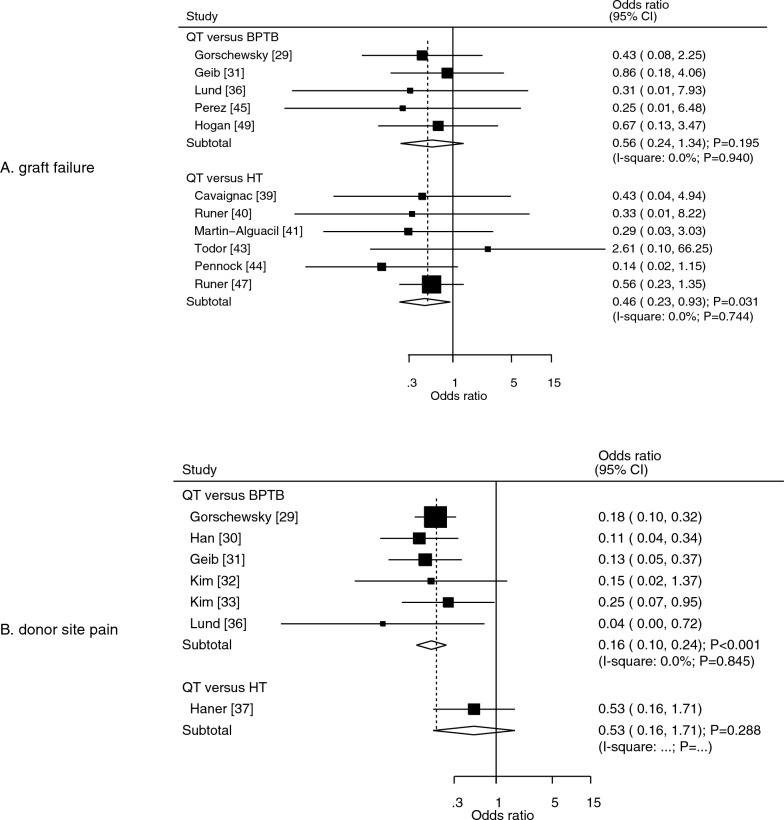
Effects of QT versus BPTB and HT autografts on graft failure (**A**) and donor-site pain (**B**).* BPTB* bone–patellar tendon–bone,* HT* hamstring tendon,* QT* quadriceps tendon

Eleven studies reported the effects of QT versus other autografts on the risk of graft failure. We noted that the the QT autograft was associated with a lower risk of graft failure than that observed with the HT autograft (OR: 0.46; 95% CI: 0.23–0.93; *P* = 0.031; Fig. 2A). The difference in the risk of graft failure between QT and BPTB autografts was not statistically significant (OR: 0.56; 95% CI: 0.24–1.34; *P* = 0.195; Fig. 2A). There was no evidence of heterogeneity across the included studies for QT versus BPTB (*I*^2^ = 0.0%; *P* = 0.940) and QT versus HT (*I*^2^ = 0.0%; *P* = 0.744). Sensitivity analyses found that the pooled conclusion regarding QT versus BPTB on the risk of graft failure was stable, whereas the conclusion regarding QT versus HT on the risk of graft failure was variable (data not shown). The subgroup analysis for the risk of graft failure according to study design is shown in Table 2, with no significant difference between QT and BPTB or between QT and HT autografts for the risk of graft failure, irrespective of whether the pooled prospective or retrospective studies were analyzed. No significant publication bias for graft failure was observed (*P* value for Egger: 0.466; *P* value for Begg: 0.276; Supplementary file 1).

**Table 2:** Subgroup analyses for reported outcomes according to study design

Outcomes	Comparisons	Subgroup	No. of studies	OR or WMD with 95% CI	*P* value	*I*^2^ (%)/*P* value	Interaction *P* value
Graft failure	QT versus BPTB	Prospective	1	0.31 (0.01–7.93)	0.477	–	0.704
		Retrospective	4	0.59 (0.24–1.45)	0.251	0.0/0.886	
	QT versus HT	Prospective	3	0.50 (0.22–1.11)	0.089	0.0/0.850	0.712
		Retrospective	3	0.38 (0.08–1.75)	0.213	11.2/0.324	
Donor site pain	QT versus BPTB	Prospective	1	0.04 (0.00–0.72)	0.029	–	0.340
		Retrospective	5	0.16 (0.10–0.25)	< 0.001	0.0/0.892	
	QT versus HT	Prospective	1	0.53 (0.16–1.71)	0.288	–	–
		Retrospective	0	–	–	–	
Lysholm score	QT versus BPTB	Prospective	0	–	–	–	–
		Retrospective	6	− 0.64 (− 1.65 to 0.37)	0.211	0.0/0.827	
	QT versus HT	Prospective	3	− 1.73 (− 4.15 to 0.68)	0.160	0.0/0.426	0.067
		Retrospective	7	1.35 (− 1.08 to 3.77)	0.276	81.6/ < 0.001	
Side-to-side difference	QT versus BPTB	Prospective	1	0.30 (− 0.56 to 1.16)	0.492	–	0.404
		Retrospective	4	− 0.24 (− 0.79 to 0.32)	0.404	76.4/0.005	
	QT versus HT	Prospective	1	− 1.00 (− 2.21 to 0.21)	0.105	–	0.679
		Retrospective	5	− 0.70 (− 1.51 to 0.12)	0.093	89.9/<0.001	
Side-to-side difference > 3	QT versus BPTB	Prospective	1	0.95 (0.26–3.47)	0.938	–	0.829
		Retrospective	6	0.74 (0.37–1.47)	0.385	73.1/0.002	
	QT versus HT	Prospective	0	–	–	–	–
		Retrospective	2	0.81 (0.40–1.65)	0.561	0.0/0.577	
Pivot-shift grade 0	QT versus BPTB	Prospective	1	9.78 (2.55–37.43)	0.001	–	0.001
		Retrospective	4	0.86 (0.51–1.47)	0.589	0.0/0.516	
	QT versus HT	Prospective	0	–	–	–	–
		Retrospective	4	1.47 (0.57–3.79)	0.424	66.9/0.028	
Lachman grade 0	QT versus BPTB	Prospective	0	–	–	–	–
		Retrospective	4	1.15 (0.57–2.32)	0.702	53.1/0.094	
	QT versus HT	Prospective	0	–	–	–	–
		Retrospective	4	1.96 (0.62–6.17)	0.251	79.8/0.002	
IKDC A or B	QT versus BPTB	Prospective	0	–	–	–	–
		Retrospective	5	0.62 (0.30–1.25)	0.178	55.0/0.064	
	QT versus HT	Prospective	1	1.61 (0.50–5.22)	0.430	–	0.538
		Retrospective	2	1.03 (0.47–2.26)	0.941	0.0/0.390	

Data on the effect of the QT autografts on the risk of donor-site pain were reported in seven studies. We noted that QT autografts were associated with a lower risk of donor-site pain than that observed with the BPTB autograft (OR: 0.16; 95% CI: 0.10–0.24; *P* < 0.001; Fig. 2B), whereas no significant difference in the risk of donor-site pain between the QT and HT autografts (OR: 0.53; 95% CI: 0.16–1.71; *P* = 0.288; Fig. 2B) was observed. No significant heterogeneity was observed for QT versus BPTB in the risk of donor-site pain (*I*^2^ = 0.0%; *P* = 0.845). Sensitivity analysis found that the pooled conclusion for QT versus BPTB on the risk of donor-site pain was stable after sequentially excluding individual studies (data not shown). The subgroup analysis for the risk of donor-site pain according to study design is shown in Table 2, and it showed that QT autografts were associated with a reduced risk of donor-site pain compared with BPTB autografts, irrespective of whether the pooled prospective or retrospective studies were analyzed (Table 2). There was no significant publication bias for donor-site pain (*P* value for Egger: 0.736; *P* value for Begg: 0.548; Supplementary file 1).

### Lysholm score and good Lysholm score ratings

**Fig. 3:**
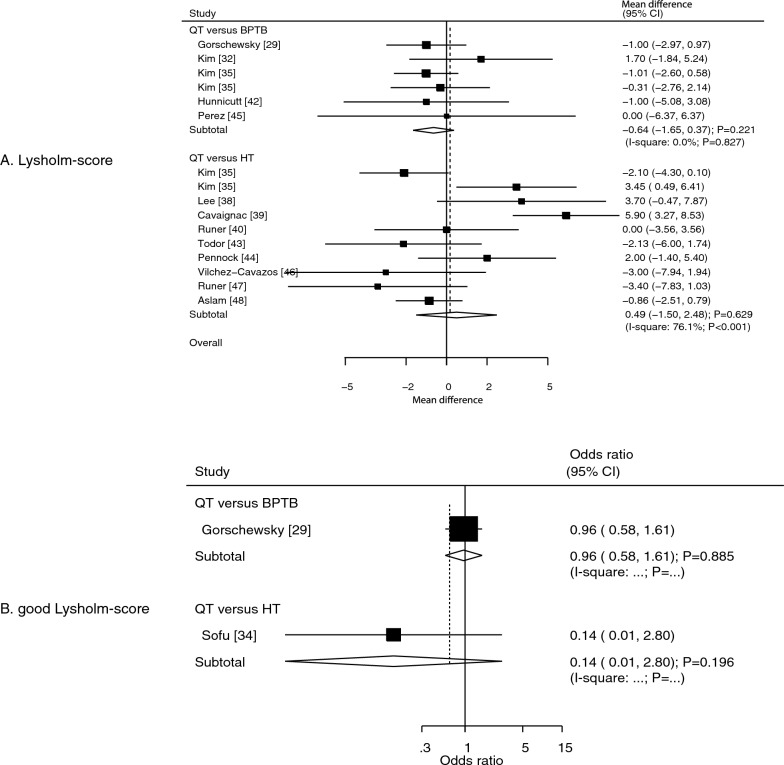
Effects of QT versus BPTB and HT autografts on the Lysholm score (**A**) and good Lysholm score ratings (**B**).* BPTB* bone–patellar tendon–bone,* HT* hamstring tendon,* QT* quadriceps tendon

Thirteen studies reported the effects of QT versus other autografts on the Lysholm score. We noted that the QT autograft was not associated with a significant difference in the Lysholm score compared to the BPTB (WMD: − 0.64; 95% CI: − 1.65 to 0.37; *P* = 0.221) or HT (WMD: 0.49; 95% CI: − 1.50 to 2.48; *P* = 0.629) autografts (Fig. 3A). There was no evidence of heterogeneity regarding QT versus BPTB in the Lysholm score (*I*^2^ = 0.0%; *P* = 0.827), while significant heterogeneity was observed for QT versus HT in the Lysholm score (*I*^2^ = 76.1%; *P* < 0.001). Sensitivity analysis indicated that the pooled conclusion was not changed by the sequential exclusion of individual studies, irrespective of whether QT was compared to BPTB or HT autografts (data not shown). The subgroup analysis for the Lysholm score according to the study design is shown in Table 2, and the results were consistent with the overall analysis (Table 2). No significant publication bias for the Lysholm score was observed (*P* value for Egger: 0.483; *P* value for Begg: 0.893; Supplementary file 1).

Data on the effect of QT autografts on achieving good Lysholm scores were reported in two studies. The QT autograft was not associated with the achievement of a good Lysholm score, irrespective of whether it was compared with BPTB (OR: 0.96; 95% CI: 0.58–1.61; *P* = 0.885) or HT (OR: 0.14; 95% CI: 0.01–2.80; *P* = 0.196) autografts (Fig. 3B).

### Side-to-side difference and side-to-side difference rating of >3

**Fig. 4:**
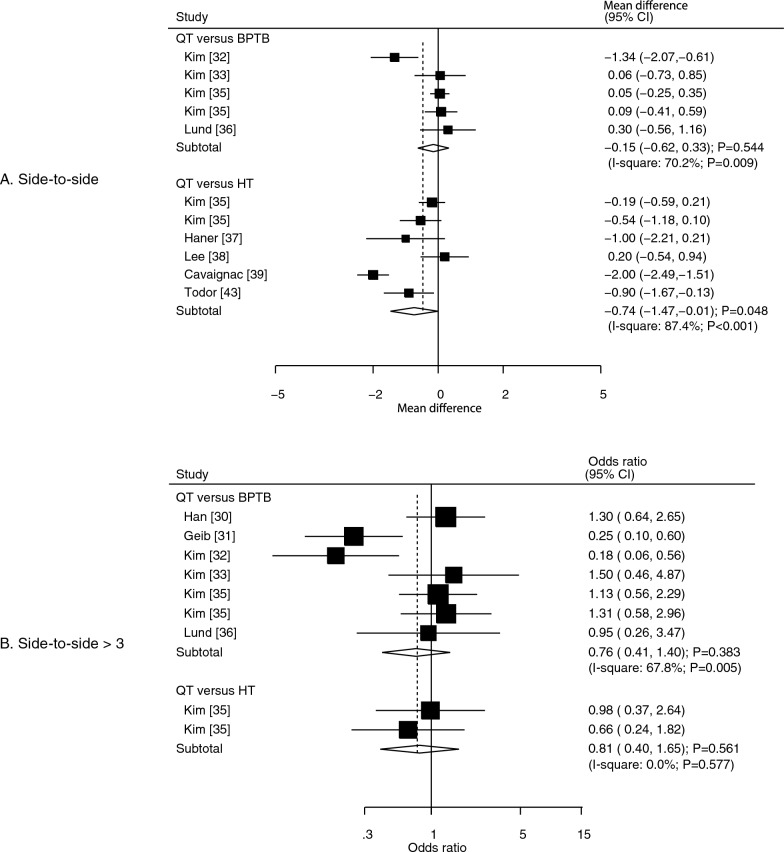
Effects of QT versus BPTB and HT autografts on side-to-side differences (**A**) and side-to-side differences > 3 (**B**).* BPTB* bone–patellar tendon–bone,* HT* hamstring tendon,* QT* quadriceps tendon

Eight studies reported side-to-side differences in the effects of the QT autograft versus other autografts. We noted that the QT autograft was associated with smaller side-to-side differences than observed with the HT autograft (WMD: − 0.74; 95% CI: − 1.47 to − 0.01; *P* = 0.048), and the side-to-side difference was not significantly different between the QT and BPTB autografts (WMD: − 0.15; 95% CI: − 0.62 to 0.33; *P* = 0.544) (Fig. 4A). Significant heterogeneity was observed across the included studies, irrespective of whether QT was compared to BPTB (*I*^2^ = 70.2%; *P* = 0.009) or to HT (*I*^2^ = 87.4%; *P* < 0.001). The pooled conclusion for QT versus BPTB was stable, while for that QT versus HT was variable owing to the marginal 95% CI (data not shown). The subgroup analysis for side-to-side differences according to study design is shown in Table 2, and no significant difference between the autografts was observed in any subgroup. There was no significant publication bias for side-to-side differences (*P* value for Egger: 0.490; *P* value for Begg: 0.755; Supplementary file 2).

Data on the effect of the QT autografts on the incidence of side-to-side differences > 3 were reported in six studies. The QT autograft was not associated with the incidence of side-to-side differences  > 3, irrespective of whether the BPTB (OR: 0.76; 95% CI: 0.41–1.40; *P* = 0.383) or HT (OR: 0.81; 95% CI: 0.40–1.65; *P* = 0.561) autografts were used as the control (Fig. 4B). There was significant heterogeneity across the included studies regarding QT versus BPTB (*I*^2^ = 67.8%; *P* = 0.005), while there was no evidence of heterogeneity for QT versus HT (*I*^2^ = 0.0%; *P* = 0.577). Sensitivity analysis indicated that the pooled conclusion was robust regarding QT versus BPTB (data not shown). The results of the subgroup analyses according to study design were consistent with the overall analysis, and the conclusions remained non-significant (Table 2). No significant publication bias for the incidence of side-to-side differences > 3 was observed (*P* value for Egger: 0.362; *P* value for Begg: 0.348; Supplementary file 1).

### Pivot-shift grade 0 and Lachman grade 0

**Fig. 5:**
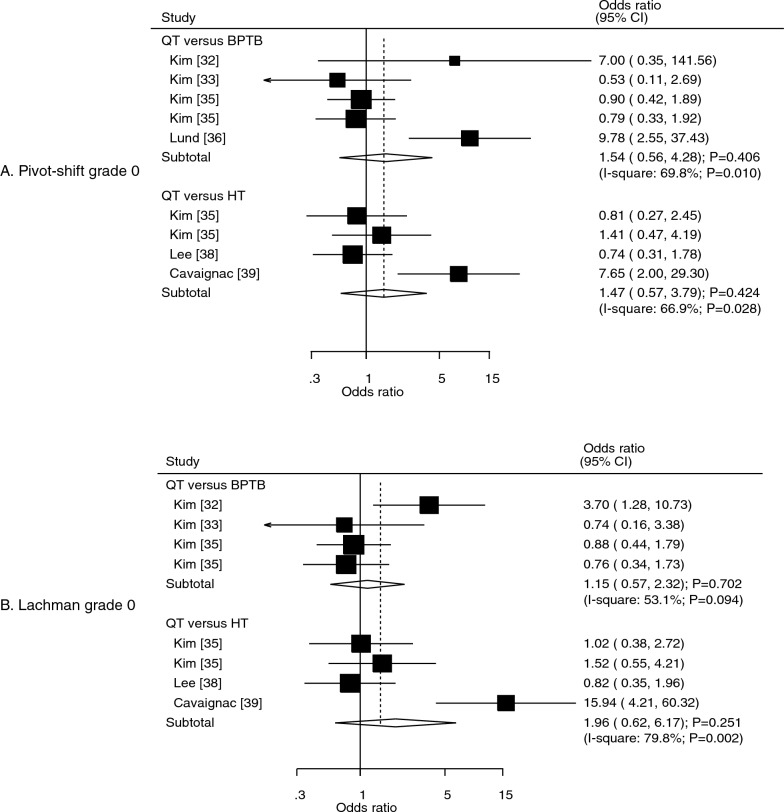
Effects of QT versus BPTB and HT autografts on the incidence of pivot-shift grade 0 (**A**) and the incidence of Lachman grade 0 (**B**).* BPTB* bone-patellar tendon-bone,* HT* hamstring tendon,* QT* quadriceps tendon

Six studies reported the effects of the QT autograft versus the other autografts on the incidence of a pivot-shift grade of 0. We noted that the QT autograft was not associated with the incidence of pivot-shift grade 0, irrespective of whether the BPTB (OR: 1.54; 95% CI: 0.56–4.28; *P* = 0.406) or HT (OR: 1.47; 95% CI: 0.57–3.79; *P* = 0.424) autograft was used as the control (Fig. 5A). There was significant heterogeneity for QT versus BPTB (*I*^2^ = 69.8%; *P* = 0.010) and for QT versus HT (*I*^2^ = 66.9%; *P* = 0.028). The subgroup analysis for the incidence of pivot-shift grade 0 according to study design is shown in Table 2. The QT autograft was associated with an increased incidence of pivot-shift grade 0 compared with the BPTB autograft (OR: 9.78; 95% CI: 2.55–37.43; *P* = 0.001). There was no significant publication bias for the incidence of pivot-shift grade 0 (*P* value for Egger: 0.142; *P* value for Begg: 0.175; Supplementary file 1).

Data on the effect of the QT autografts on the incidence of Lachman grade 0 were reported in five studies. The QT autograft was not associated with the incidence of Lachman grade 0, irrespective of whether the BPTB (OR: 1.96; 95% CI: 0.62–6.17; *P* = 0.251) or HT (OR: 1.15; 95% CI: 0.57–2.32; *P* = 0.702) autograft was used as the control (Fig. 5B). There was significant heterogeneity for QT versus BPTB (*I*^2^ = 53.1%; *P* = 0.094) and for QT versus HT (*I*^2^ = 79.8%; *P* = 0.002). The results of the subgroup analyses according to study design were consistent with the overall analysis, and the conclusions remained non-significant (Table 2). Although the Egger test indicated no significant publication bias (*P* = 0.144), the Begg test suggested a potential publication bias for the incidence of Lachman grade 0 (*P* = 0.035; Supplementary file 1). The conclusion was not altered after adjusting for potential publication bias using the trim-and-fill method [50].

### International Knee Documentation Committee Subjective Knee Form grade A or B, subjective satisfaction, and moderate-to-severe symptoms during sports and work

**Fig. 6:**
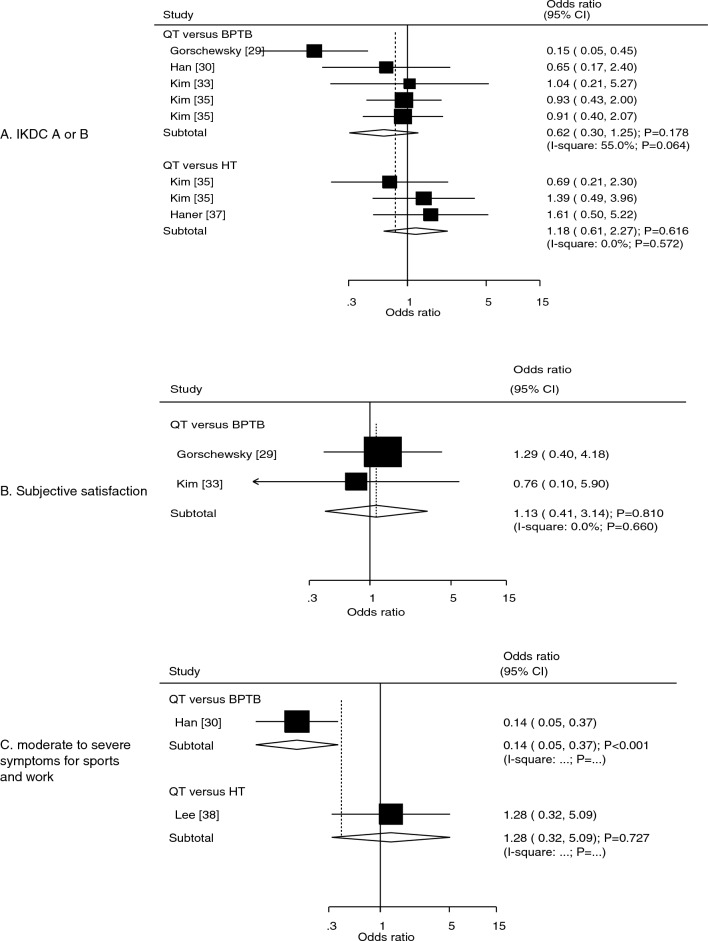
Effects of QT versus BPTB and HT autografts on the incidence of IKDC A or B (**A**), on subjective satisfaction (**B**), and on the incidence of moderate-to-severe kneecap symptoms during sports and work activities (**C**).* BPTB* bone–patellar tendon–bone,* HT* hamstring tendon,* QT* quadriceps tendon

Five studies reported the effects of the QT versus other autografts on the incidence of International Knee Documentation Committee (IKDC) Subjective Knee Form grade A or B. The QT autograft did not affect the incidence of IKDC A or B compared with that observed with the BPTB (OR: 0.62; 95% CI: 0.30–1.25; *P* = 0.178) or HT (OR: 1.18; 95% CI: 0.61–2.27; *P* = 0.616) autograft (Fig. 6A). We noted potential heterogeneity for QT versus BPTB (*I*^2^ = 55.0%; *P* = 0.064), while there was no evidence of heterogeneity for QT versus HT (*I*^2^ = 0.0%; *P* = 0.572). The results of the subgroup analyses according to study design were consistent with the overall analysis, and the conclusions remained non-significant (Table 2). No significant publication bias for the incidence of IKDC A or B was observed (*P* value for Egger: 0.808; *P* value for Begg: 0.536; Supplementary file 1).

Data on the effect of the QT autografts on subjective satisfaction were reported in two studies. There was no significant difference between the QT and BPTB autografts in terms of subjective satisfaction (OR: 1.13; 95% CI: 0.41–3.14; *P* = 0.810; Fig. 6B) and no evidence of heterogeneity across the included studies (*I*^2^ = 0.0%; *P* = 0.660). Moreover, two studies reported the effect of the QT autograft on the incidence of moderate-to-severe symptoms during sports and work activities. The QT autograft was associated with a lower incidence of moderate-to-severe kneecap symptoms during sports and work activities compared with the BPTB autograft (OR: 0.14; 95% CI: 0.05–0.37; *P* < 0.001). However, no significant difference was observed between the QT and HT autografts in the incidence of moderate-to-severe kneecap symptoms during sports and work activities (OR: 1.28; 95% CI: 0.32–5.09; *P* = 0.727) (Fig. 6C).

## Discussion

This updated systematic review and meta-analysis identified 2964 patients with ACLR from 21 studies with a broad range of patient characteristics. This study found that QT autografts were associated with a lower risk of donor-site pain and moderate-to-severe symptoms during sports and work activities versus BPTB autografts. Moreover, the QT autografts were associated with a lower risk of graft failure and smaller side-to-side differences versus HT autografts. Furthermore, QT autografts were associated with an increased incidence of pivot-shift grade 0 versus BPTB autografts in the pooled prospective studies.

Several systematic reviews and meta-analyses have compared the efficacy and safety of various autografts for ACLR [51–56]. A Bayesian network meta-analysis conducted by Migliorini et al. identified 2,603 knees and found that the QT autograft was a feasible option for primary ACLR [51]. Zhou et al. identified 15 studies and found that the BPTB autograft was more associated with an increased risk of contralateral ACL rupture than the HT autograft was [52]. Bergeron et al. identified 29 studies and found no significant difference between the BPTB and HT autografts in the return to baseline physical activity and/or sports participation level [53]. A meta-analysis conducted by Hurley et al. included 15 studies and suggested that the QT autograft was associated with a lower risk of re-rupture and donor-site morbidity compared with the HT autograft. Moreover, the QT autograft showed a better residual pivot shift versus the HT autograft [54]. Herbawi et al. identified 10 studies and found that QT autografts showed better results for knee flexion versus HT autografts, whereas the QT and BPTB autografts gave similar results. Moreover, the use of an HT autograft was associated with superior results regarding knee extension compared with the QT autograft [55]. Dai et al. performed a meta-analysis of 24 studies and found QT showed comparable graft survival, functional outcomes, and stability outcomes as compared with HT and BPTB. Moreover, QT was associated with a lower risk of donor-site morbidity [56]. However, considering that the inclusion criteria across previous studies were not consistent, and several important results regarding the use of the QT, BPTB, and HT autografts for ACLR were not investigated, the present updated systematic review and meta-analysis was performed to determine the treatment effects of QT, BPTB, and HT autografts for ACLR.

In summary, our results suggest that the QT autografts showed a lower risk of donor-site pain and moderate-to-severe symptoms during sports and work activities versus BPTB autografts. A potential reason for this could be that the QT autograft is longer and thicker than the BPTB autograft and attaches to the patella more widely [57]. Moreover, the QT autograft was associated with a larger cross-sectional area than that covered by the patellar tendon, whereas the ultimate tensile stress and strain of the patellar tendon were larger than those of the QT autograft [58]. Furthermore, several factors such as paresthesia, anterior knee pain, incision size, and scar cosmesis could affect the incidence of donor-site morbidity. Specifically, hypoesthesia from nerve injuries is significantly related to patient dissatisfaction and donor-site complaints [59, 60]. Finally, although the risk of moderate-to-severe symptoms during sports and work activities are lower in patients in whom a QT autograft rather than an HT or BPTB autograft was used for ACLR, only two studies reported these results, and the pooled conclusion might vary.

We noted that the QT autograft was associated with a lower risk of graft failure and smaller side-to-side differences than those observed with the HT autograft. Potential reasons for these include graft thickness, variation in the HT configuration, the number of strands, and the method of graft fixation. Moreover, a patellar bone block was applied in the QT harvesting technique used in most of the included studies, and bone-to-bone healing was superior to tendon-to-bone healing, which was associated with a lower risk of graft re-rupture [61, 62].

The functional outcomes of the QT, BPTB, and HT autografts were comparable, whereas the subgroup analysis found that the QT autografts were associated with an increased incidence of pivot-shift grade 0 versus BPTB autografts in the pooled prospective studies. However, this result was only reported in one study [36] and was adequately explained by a QT autograft with a larger cross-sectional area in the intra-articular portion of an ACLR [63]. Moreover, although the results of the subgroup analyses for the investigated outcomes were obtained according to study design, most of the included studies were retrospective observational studies; thus, further prospective studies are required.

An important point to raise is that there are two distinct methods for harvesting and implanting the QT in ACLR surgery: ribbon-shaped QT harvesting with square tunnel reconstruction and circular QT harvesting implemented through cylindrical tunnels, mimicking the patellar tendon or hamstring techniques. Ribbon-shaped harvesting typically preserves the natural fiber arrangement of the QT, which may more closely resemble the native anatomy and function of the ACL. This approach is likely to facilitate the restoration of knee joint stability and range of motion. Square tunnels provide a larger surface area for contact, which is conducive to tendon-to-bone healing and enhances the initial stability of the reconstructed ligament. Harvesting the circular QT and replicating the reconstruction style of the patellar tendon or hamstring may place greater emphasis on the central stability of the reconstructed ligament and minimizing interference with surrounding tissues. This approach might be better suited for those seeking specific mechanical properties or accommodating certain patient anatomies. Square tunnel reconstruction demands higher precision and surgical skill to ensure accurate tunnel positioning and sizing as well as secure fixation of the ribbon-shaped tendon, thus avoiding postoperative complications such as displacement or rupture. In contrast, cylindrical tunnels offer potentially simpler technical procedures, reducing surgical complexity, and existing experiences from patellar tendon or hamstring reconstructions can be leveraged to expedite the surgical process. Future studies should compare the efficacy and safety of these two distinct methods of harvesting and implanting the QT in ACLR surgeries.

This study has several limitations. First, the analysis included RCTs, prospective observational studies, and retrospective observational studies, and the results could have been affected by recall and confounder biases. Second, the prognosis of patients after ACLR differs between the skeletally immature and mature, which could affect the treatment effects of the QT, BPTB, and HT autografts for ACLR [64]. Third, the therapeutic outcomes of using QT, BPTB, and HT autografts for ACLR may be influenced by the necessity for revisions or alternative procedures following the initial implementation of either technique, particularly concerning the overall differences between these approaches. Fourth, a small number of studies reported several outcomes, and the pooled conclusion was variable. Fifth, the heterogeneity across the included studies was substantial for several reported outcomes, which was not fully explained by the sensitivity and subgroup analyses. The potential reason for this could be the various characteristics of the patients and rehabilitation procedures among the included studies. Moreover, the widely variable follow-up times and the absence of detailed demographic data could be considered a potential source of heterogeneity. Finally, there are inherent limitations to the meta-analysis of published articles, including inevitable publication bias and restricted detailed analyses.

## Conclusion

The findings of this study indicated that, compared with the BPTB autograft, the QT autograft was associated with a lower risk of donor-site pain and moderate-to-severe kneecap symptoms during sports and work activities. Furthermore, compared with HT autografts, QT autografts were associated with a lower risk of graft failure and fewer side-to-side differences. Further large-scale RCTs should be performed to compare the efficacy and safety of the QT autograft versus the BPTB and HT autografts for ACLR according to patient characteristics.

## Supplementary information

Supplementary file 1.

Supplementary file 2.

## Data Availability

All relevant data are included within the paper and its supplementary information files.

## Abbreviations

ACL: Anterior cruciate ligament
ACLR: Anterior cruciate ligament reconstruction
BPTB: Bone–patellar tendon–bone
HT: Hamstring tendon
QT: Quadriceps tendon
PRISMA: Preferred Reporting Items for Systematic Reviews and Meta-analysis
PICOS: Patients, intervention, control, outcomes, and study design
RCTs: Randomized controlled trials
NOS: Newcastle–Ottawa Scale
OR: Odds ratios
WMD: Weighted mean differences
CI: Confidence intervals
IKDC: International Knee Documentation Committee
