# Supplementary material for: Efficacy and safety of quadriceps tendon autograft versus bone–patellar tendon–bone and hamstring tendon autografts for anterior cruciate ligament reconstruction: a systematic review and meta-analysis
Source: J Orthop Traumatol. 2024 Dec 18;25:65. doi: 10.1186/s10195-024-00801-2 (PMC11656020; doi:10.1186/s10195-024-00801-2)
Supplement: Supplementary file 1 [file 10195_2024_801_MOESM1_ESM.docx]

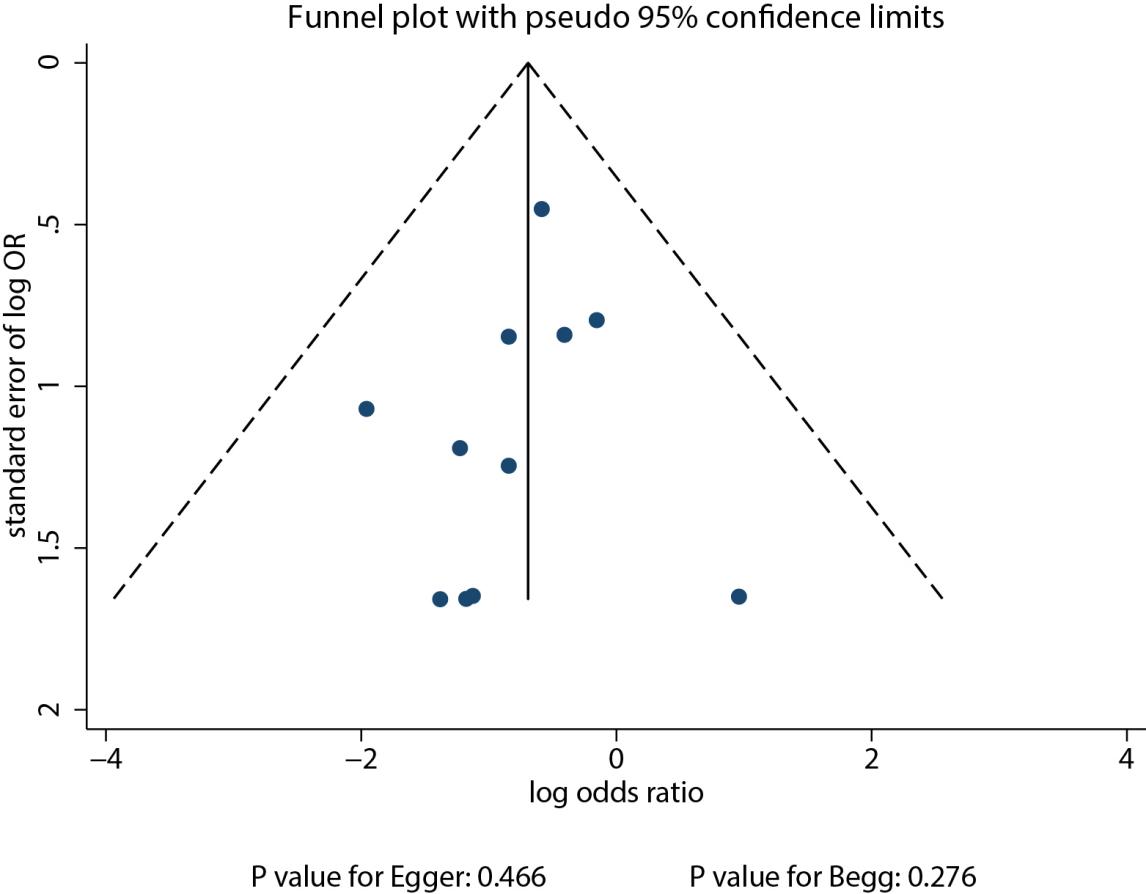


Figure S1. Funnel plot for graft failure


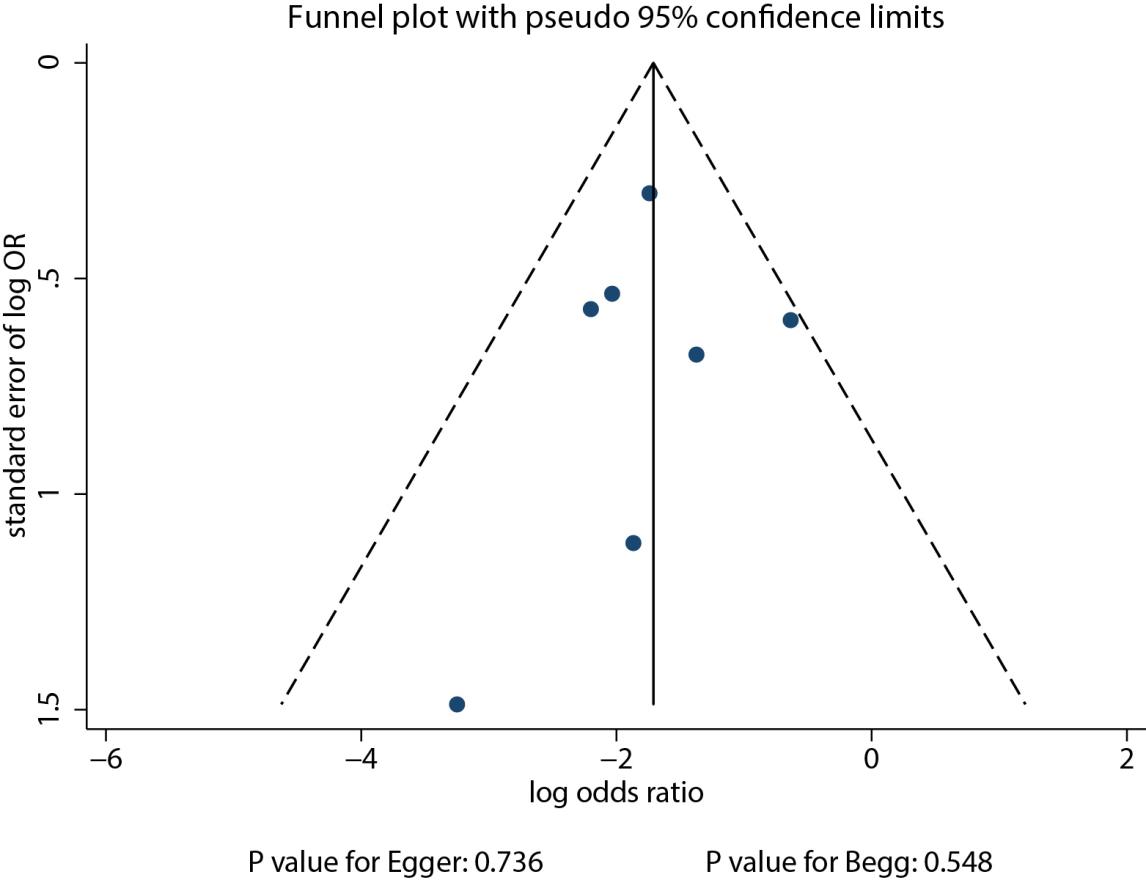


Figure S2. Funnel plot for donor site pain


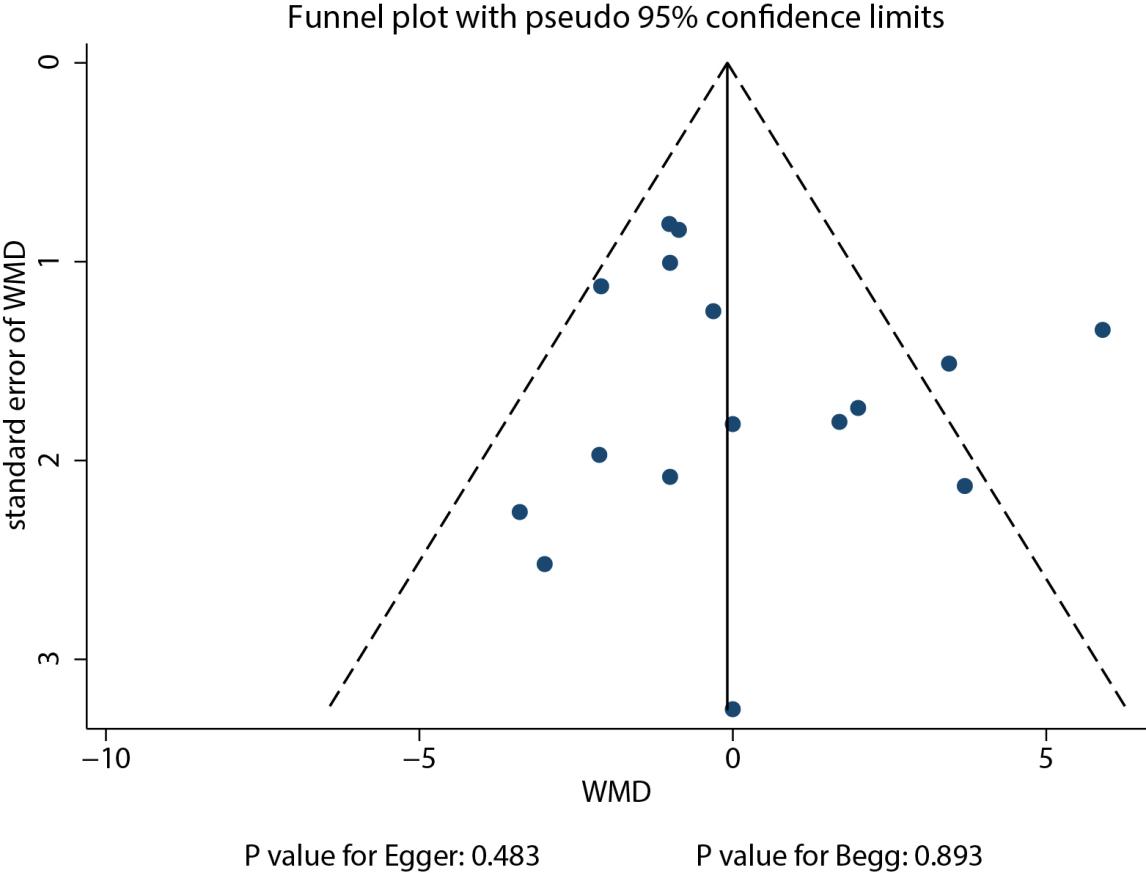


Figure S3. Funnel plot for Lysholm score


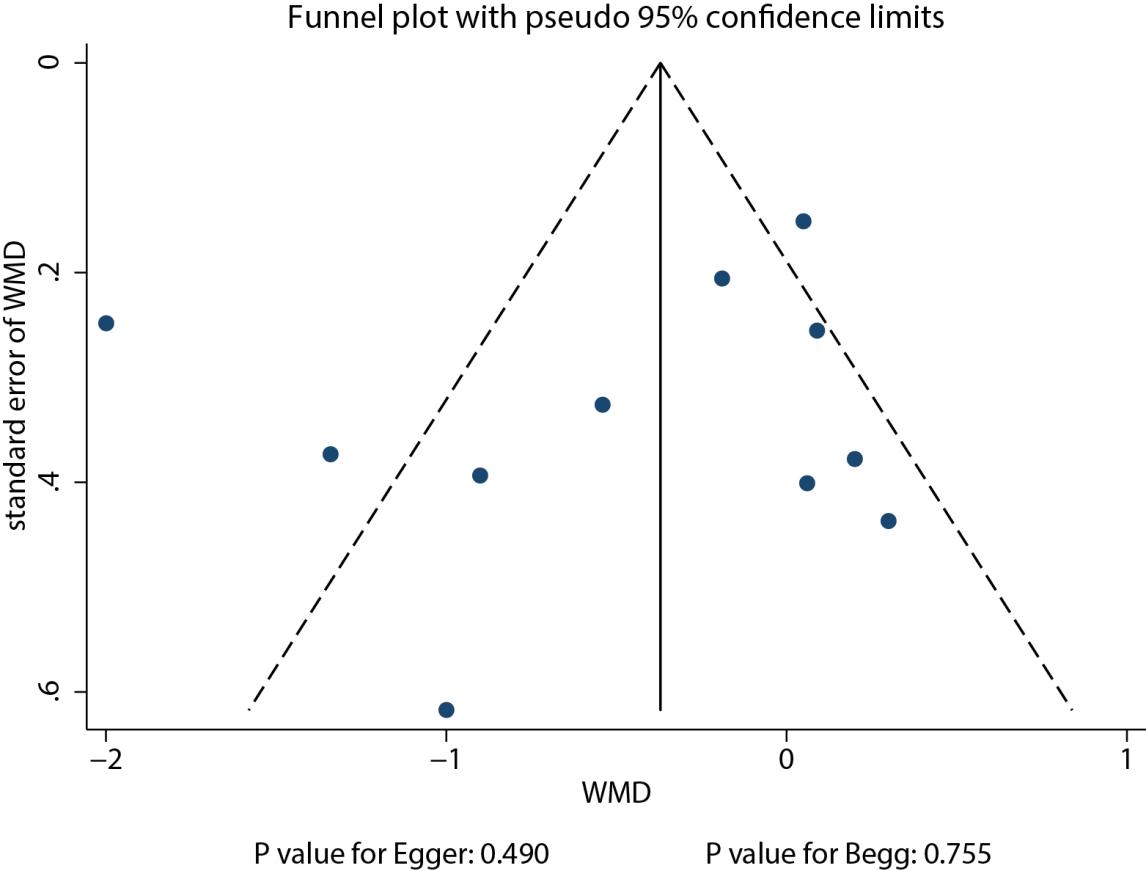


Figure S4. Funnel plot for side-to-side differences


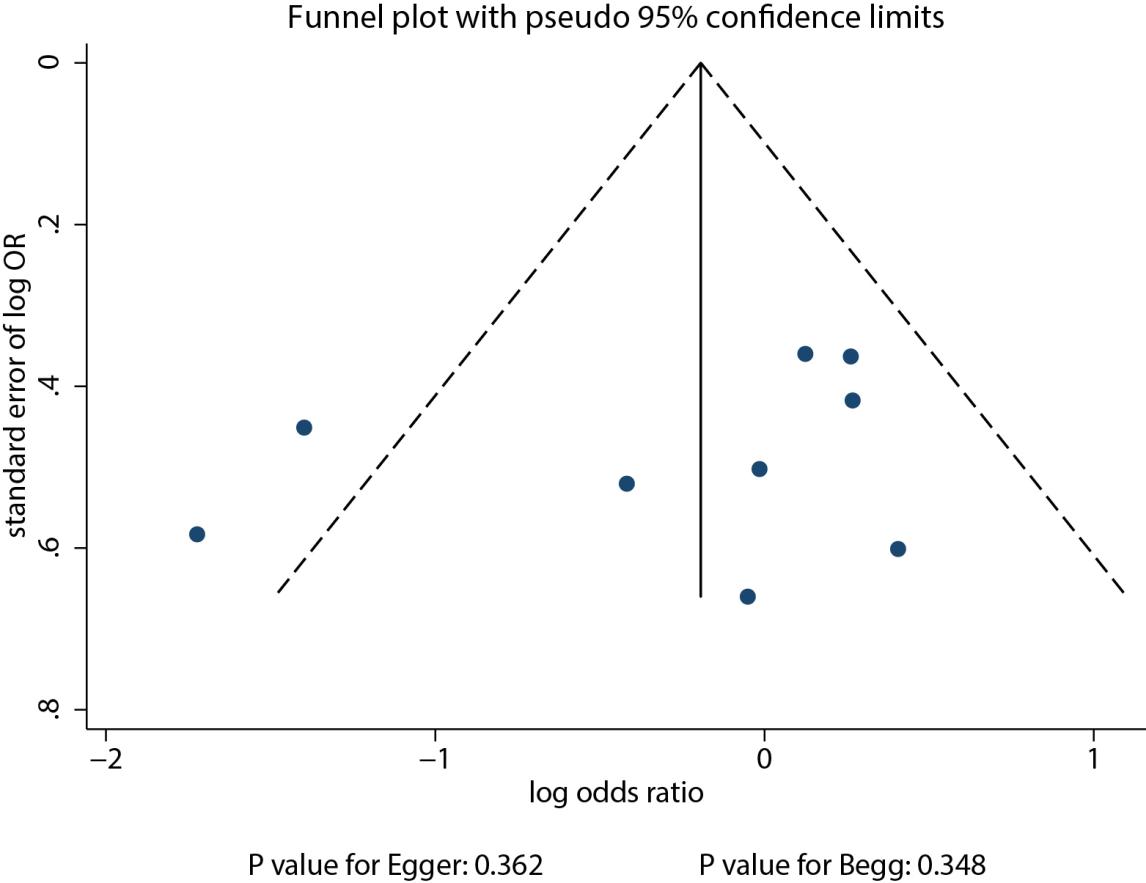


Figure S5. Funnel plot for side-to-side differences>3


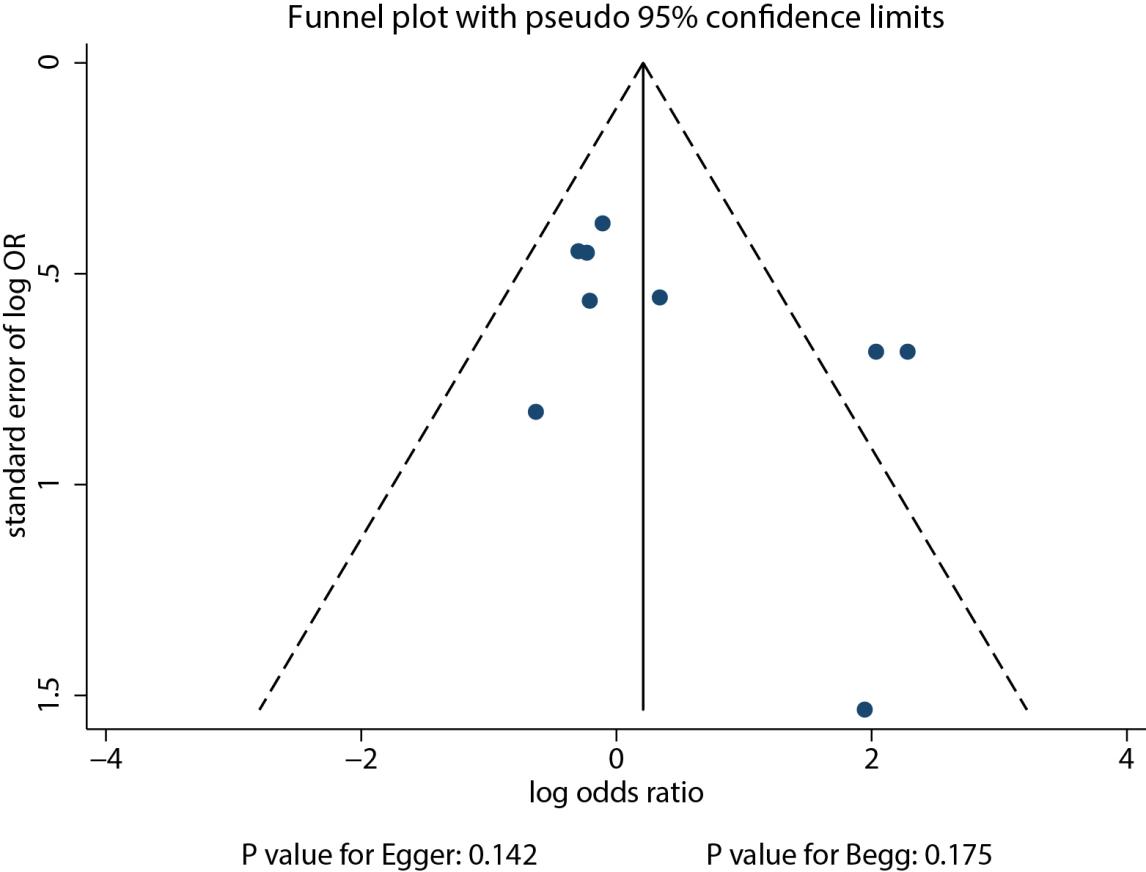


Figure S6. Funnel plot for pivot-shift grade of 0


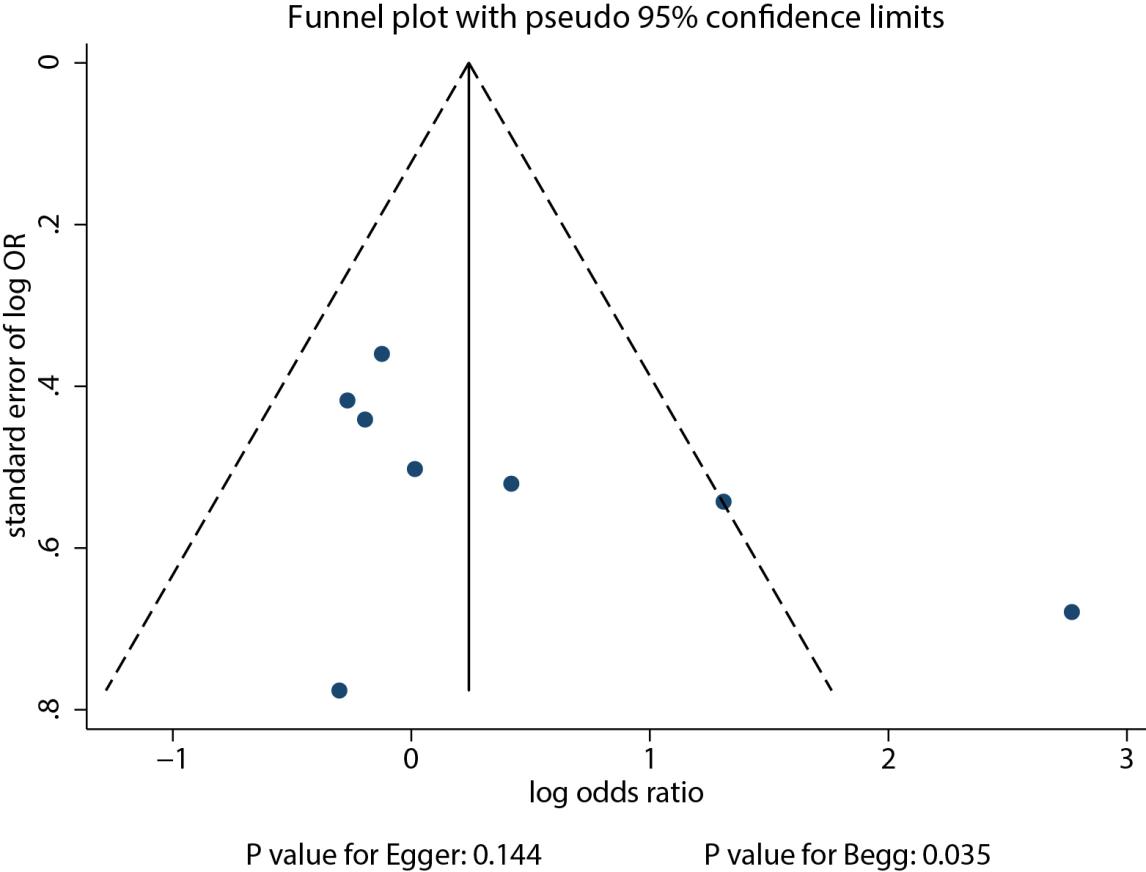


Figure S7. Funnel plot for Lachman grade 0


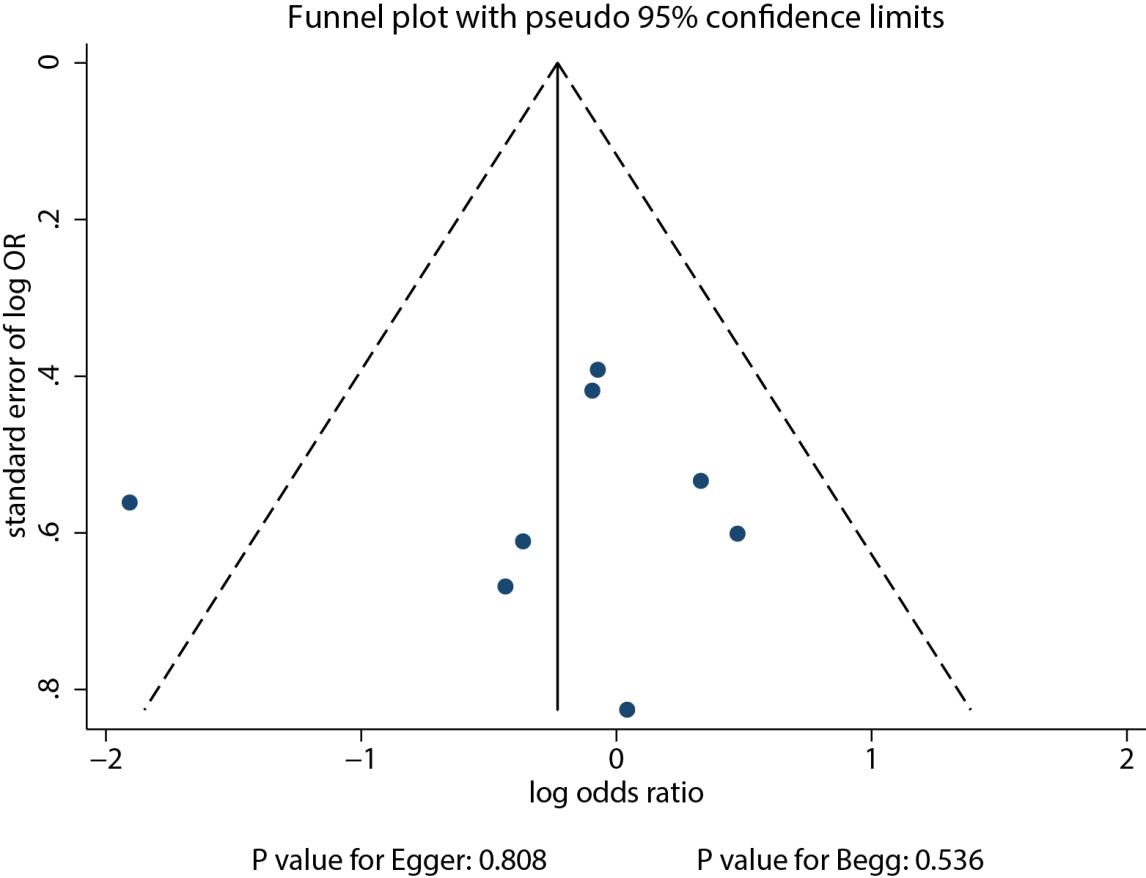


Figure S8. Funnel plot for IKDC grade A or B
